# *Commelina communis* Mitigates FB1-Induced Apoptotic Responses in Porcine Intestinal Epithelial Cells

**DOI:** 10.3390/toxins18100445

**Published:** 2026-10-15

**Authors:** Yo Han Lee, Ye Rin Bae, Tae Hong Kang, Jung Woong Yoon, Seung Joon Lim, Chae Hyun Lee, Suhyeon Yun, Sang In Lee

**Affiliations:** 1 Department of Animal Science and Biotechnology, Kyungpook National University, Sangju-si 37224, Gyeongsangbuk-do, Republic of Korea; leeyohan0216@gmail.com (Y.H.L.); apflshdk303@gmail.com (Y.R.B.); camwuil27@knu.ac.kr (T.H.K.); kizx789@knu.ac.kr (J.W.Y.); coocu1214@gmail.com (S.J.L.); dlcogus9602@naver.com (C.H.L.); ysh101612@gmail.com (S.Y.); 2 Research Institute for Innovative Animal Science, Kyungpook National University, Sangju-si 37224, Gyeongsangbuk-do, Republic of Korea

**Keywords:** fumonisin B1, apoptosis, Gene Ontology, natural products

## Abstract

Fumonisin B1 (FB1) is a mycotoxin commonly found in animal feed, particularly in corn-based products. Exposure to FB1 can damage multiple organs, including the intestine, but its molecular effects on intestinal epithelial cells remain incompletely understood. This study investigated FB1-induced toxicity in porcine intestinal epithelial (IPEC-J2) cells and screened natural products for protective effects. Exploratory gene expression profiling identified genes showing predefined expression changes following FB1 exposure, and subsequent Gene Ontology (GO) and Kyoto Encyclopedia of Genes and Genomes (KEGG) enrichment analyses indicated associations with apoptosis-related processes. Annexin V/propidium iodide staining showed increased apoptosis-associated staining, while quantitative real-time polymerase chain reaction (qRT-PCR) showed altered expression of *BCL2*, *CASP3*, and *CASP9*. To identify protective candidates, 332 natural products were evaluated using high-throughput screening. Among the screened extracts, *C. communis* showed the highest cell viability in the primary screening and was selected for further investigation. Co-treatment with *C. communis* was associated with reduced apoptosis-associated staining and partial counteraction of FB1-associated changes in apoptosis-related gene expression. These findings suggest that *C. communis* warrants further investigation as a natural product for mitigating FB1-associated intestinal epithelial toxicity.

## 1. Introduction

Mycotoxins are toxic secondary metabolites produced by fungi that can compromise the safety of food and feed and pose health risks to both humans and animals. Among the various mycotoxins identified, fumonisin B1 (FB1) is of particular concern. It is produced by *Fusarium verticillioides*, previously classified as *Gibberella fujikuroi* var. *moniliformi* [1,2,3]. FB1 commonly contaminates corn and corn-derived products. This contamination is particularly relevant to the swine industry, in which corn is widely used as a major dietary component [4,5]. Exposure to FB1 has been associated with various toxic effects, including immunotoxicity, organ toxicity, and reproductive toxicity, in both humans and animals [6,7,8].

Mechanistically, FB1 disrupts sphingolipid metabolism by inhibiting ceramide synthase, resulting in altered membrane stability, signal transduction, and apoptosis-related responses. Additionally, FB1 has been reported to downregulate the expression of TLR4 and MyD88, thereby affecting inflammatory responses [5,9]. These effects suggest that FB1 interferes with vital processes required for maintaining intestinal epithelial function.

The intestinal epithelium is one of the first tissues exposed to ingested mycotoxins and is therefore particularly susceptible to their toxic effects [10]. Intestinal epithelial cells play a major role in the absorption of essential nutrients [11]. They also prevent the translocation of harmful substances, such as bacteria, parasites, and toxins, into the body [10]. Damage to epithelial cells disrupts immune homeostasis and barrier function, thereby increasing the risk of infection and gastrointestinal disease [12]. Therefore, maintaining epithelial integrity is essential for gut health. Several studies have demonstrated that FB1 interferes with the maturation of intestinal epithelial cells, alters the expression levels of tight junction (TJ) proteins, and triggers both oxidative and endoplasmic reticulum (ER) stress, ultimately compromising intestinal barrier integrity [9,13]. However, the molecular responses of intestinal epithelial cells to FB1 exposure remain incompletely understood and require further investigation.

Various strategies, including physical, chemical, and biological approaches, have been developed to reduce mycotoxin contamination in feed. However, these methods often have limitations, such as low efficiency and nutrient loss or degradation [14,15,16]. To overcome these limitations, recent studies have investigated the use of natural products to mitigate the adverse effects of mycotoxins [17]. *Commelina communis* is an annual herb widely distributed in East Asia and has been demonstrated to possess anti-inflammatory, antioxidant, and antimicrobial properties [18,19]. Based on these reported activities, *C. communis* may have the potential to alleviate cellular damage associated with mycotoxin exposure. However, whether *C. communis* can alleviate FB1-induced toxicity in intestinal epithelial cells remains unclear.

In the present study, we investigated the effects of FB1 on porcine intestinal epithelial (IPEC-J2) cells using transcriptomic analysis and evaluated apoptosis-associated cellular responses. In addition, a library of natural product extracts was screened to identify candidates capable of mitigating FB1-induced toxicity, followed by further evaluation of selected extracts. This study aimed to improve our understanding of FB1-associated intestinal epithelial toxicity and to assess natural products as potential modulators of FB1-induced cellular injury. By integrating exploratory transcriptomic profiling with high-throughput screening of 332 natural product extracts and secondary validation of selected candidates, this study provides a systematic approach for identifying potential natural modulators of FB1-induced intestinal epithelial injury.

## 2. Results

### 2.1. Fumonisin B1 Exhibits Cytotoxic Effects on IPEC-J2 Cells

**Figure 1:**
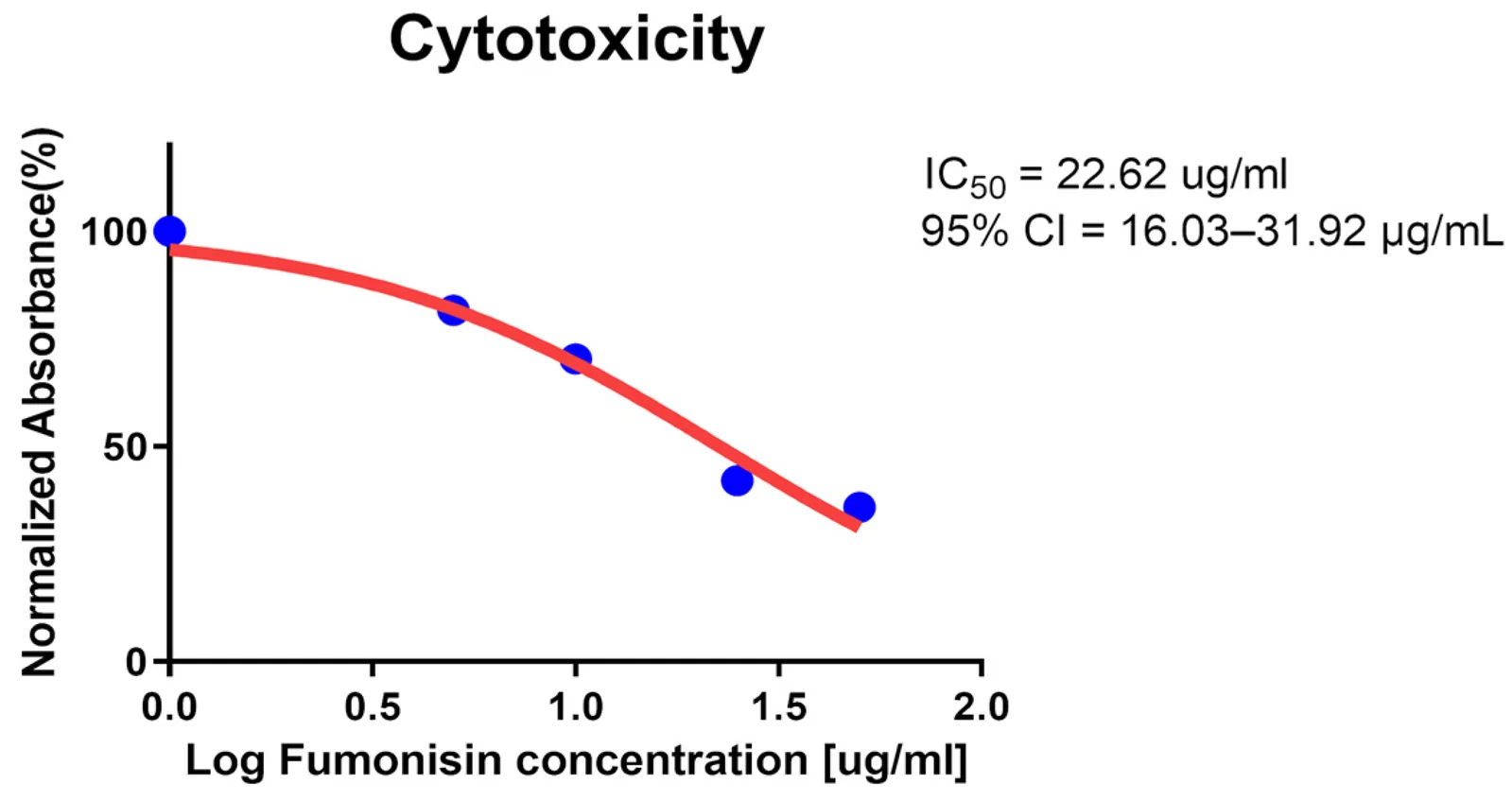
Effect of FB1 on IPEC-J2 cell viability. Cells were treated with FB1 (0–50 μg/mL) for 48 h, and viability was assessed using the WST-1 assay. Symbols represent mean values from six independent experiments (n = 6).

To evaluate the effect of FB1 on cell viability, IPEC-J2 cells were treated with FB1 at concentrations of 0, 5, 10, 25, and 50 µg/mL for 48 h, followed by WST-1 analysis (Figure 1). The estimated IC50 value was 22.62 µg/mL, with a 95% confidence interval (CI) of 16.03–31.92 µg/mL. Based on this value, 25 µg/mL FB1 was selected for subsequent experiments.

### 2.2. Exploratory Analysis and Validation of FB1-Associated Gene Expression Changes

**Figure 2:**
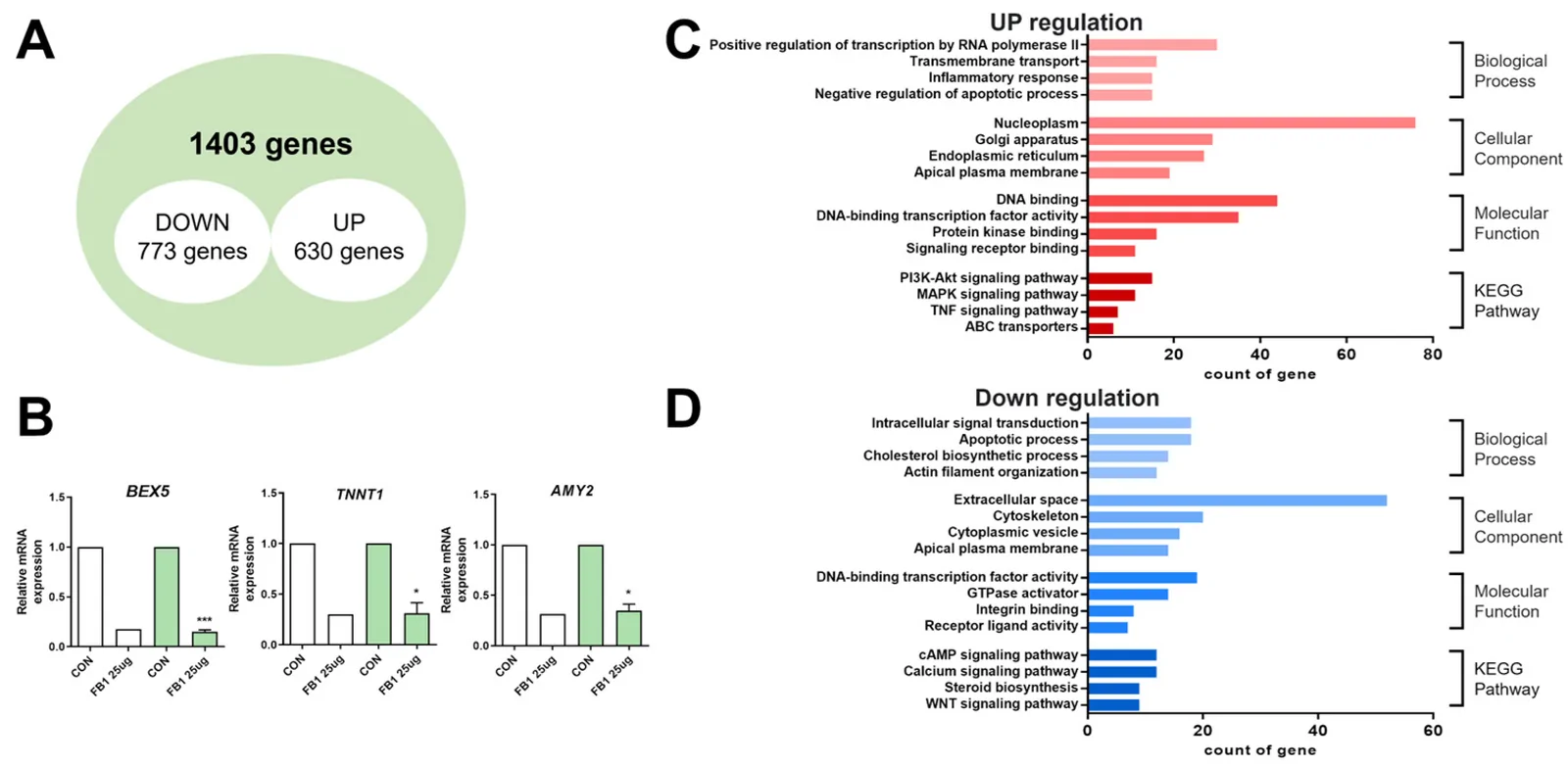
Exploratory gene expression profiling of FB1-treated IPEC-J2 cells. (**A**) Candidate genes meeting the predefined criterion, including 773 genes with decreased expression and 630 genes with increased expression. (**B**) qRT-PCR validation of *BEX5*, *TNNT1*, and *AMY2*. RNA-seq was performed using one biological sample per condition, whereas qRT-PCR data are presented as the mean ± SEM from three independent experiments (* *p* < 0.05, *** *p* < 0.001). (**C**,**D**) GO and KEGG enrichment analyses of upregulated (**C**) and downregulated (**D**) candidate genes.

IPEC-J2 cells were treated with 25 μg/mL FB1 for 48 h, followed by exploratory gene expression profiling. Based on the predefined fold-change criterion, a total of 1403 genes showed altered expression following FB1 treatment, including 630 genes with a ≥1.5-fold increase and 773 genes with a ≤0.67-fold decrease (Figure 2A). Among the genes showing decreased expression, *BEX5*, *TNNT1*, and *AMY2* exhibited prominent expression changes and were further validated by quantitative real-time polymerase chain reaction (qRT-PCR) in independent experiments (Figure 2B). Gene Ontology (GO) enrichment analysis of the 630 upregulated candidate genes revealed several enriched terms across three categories: Biological Process, Cellular Component, and Molecular Function. In the Biological Process category, the enriched terms included “positive regulation of transcription by RNA polymerase II,” “transmembrane transport,” “inflammatory response,” and “negative regulation of apoptotic process.” In the Cellular Component category, “nucleoplasm,” “Golgi apparatus,” “endoplasmic reticulum,” and “apical plasma membrane” were identified. In the Molecular Function category, the enriched terms included “DNA binding,” “DNA-binding transcription factor activity,” “protein kinase binding,” and “signaling receptor binding.” Kyoto Encyclopedia of Genes and Genomes (KEGG) pathway analysis of the upregulated candidate genes identified “PI3K-Akt signaling pathway,” “MAPK signaling pathway,” “TNF signaling pathway,” and “ABC transporters” (Figure 2C). GO enrichment analysis of the 773 downregulated candidate genes identified “intracellular signal transduction,” “apoptotic process,” “cholesterol biosynthetic process,” and “actin filament organization” in the Biological Process category. In the Cellular Component category, “extracellular space,” “cytoskeleton,” “cytoplasmic vesicle,” and “apical plasma membrane” were identified. In the Molecular Function category, the enriched terms included “DNA-binding transcription factor activity,” “GTPase activator,” “integrin binding,” and “receptor ligand activity.” KEGG pathway analysis of the downregulated candidate genes identified “cAMP signaling pathway,” “calcium signaling pathway,” “steroid biosynthesis,” and “WNT signaling pathway” (Figure 2D).

### 2.3. Fumonisin B1 Induces Apoptosis-Associated Responses in IPEC-J2 Cells

**Figure 3:**
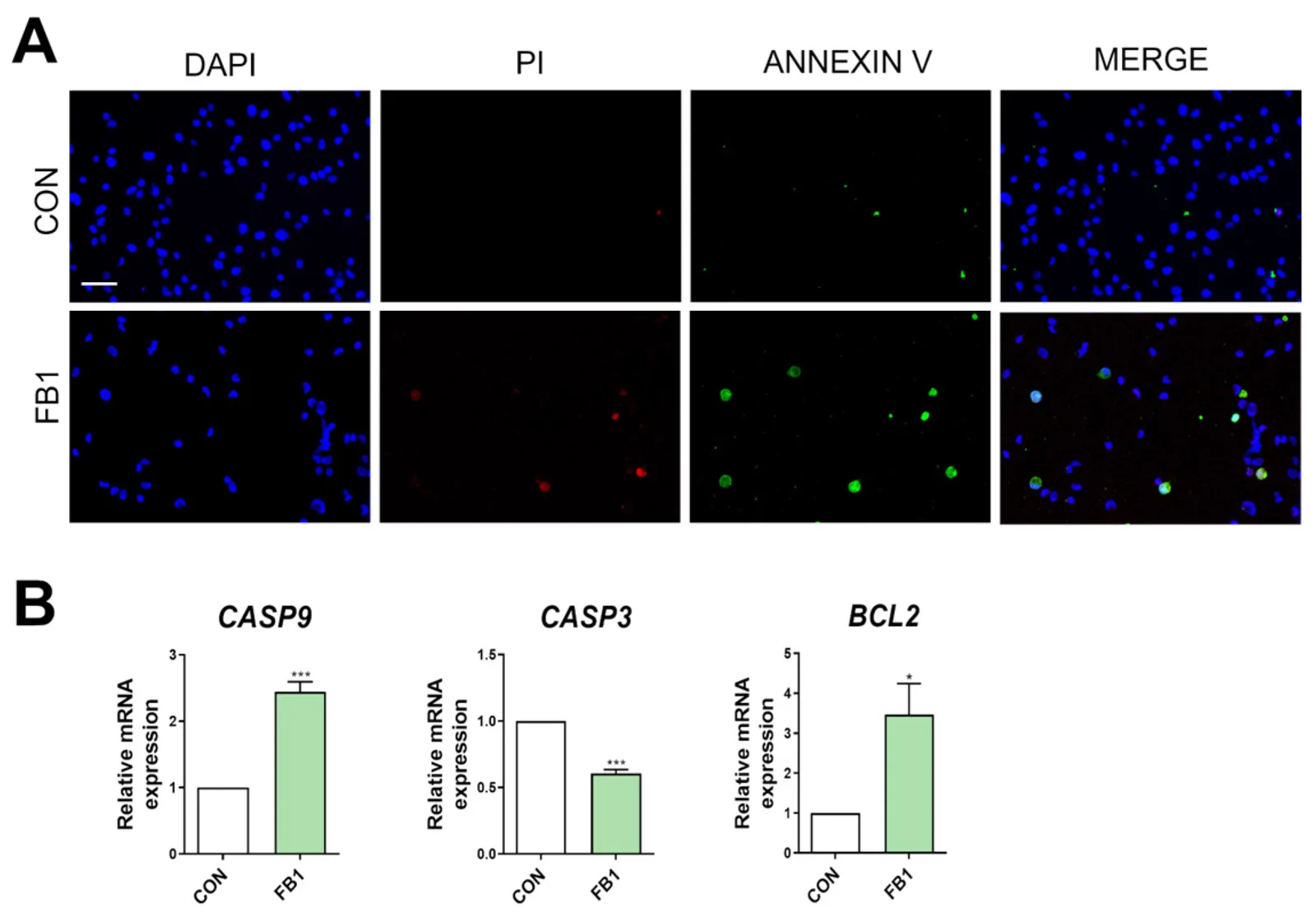
Apoptosis-associated responses in IPEC-J2 cells following FB1 exposure. (**A**) Representative Annexin V/PI fluorescence images. Scale bar = 150 μm. (**B**) mRNA expression of *CASP9*, *CASP3*, and *BCL2*. Data are presented as the mean ± SEM from three independent experiments (* *p* < 0.05, *** *p* < 0.001).

Enrichment analysis of GO terms indicated that apoptosis-related processes were associated with FB1 exposure in IPEC-J2 cells. To further examine apoptosis-associated changes, Annexin V/PI staining was performed. FB1-treated cells showed more prominent Annexin V/PI-positive staining than control cells (Figure 3A). In parallel, the mRNA expression levels of the apoptosis-related genes *CASP9*, *CASP3*, and *BCL2* were analyzed by quantitative real-time polymerase chain reaction (qRT-PCR). FB1 treatment resulted in significant alterations in the expression of *CASP9* (*p* = 0.0006), *CASP3* (*p* = 0.0002), and *BCL2* (*p* = 0.0328) compared with the control group (Figure 3B). Together, these findings support an association between FB1 exposure and apoptotic responses in IPEC-J2 cells.

### 2.4. High-Throughput Screening of Natural Products Against FB1-Induced Cytotoxicity in IPEC-J2 Cells

**Figure 4:**
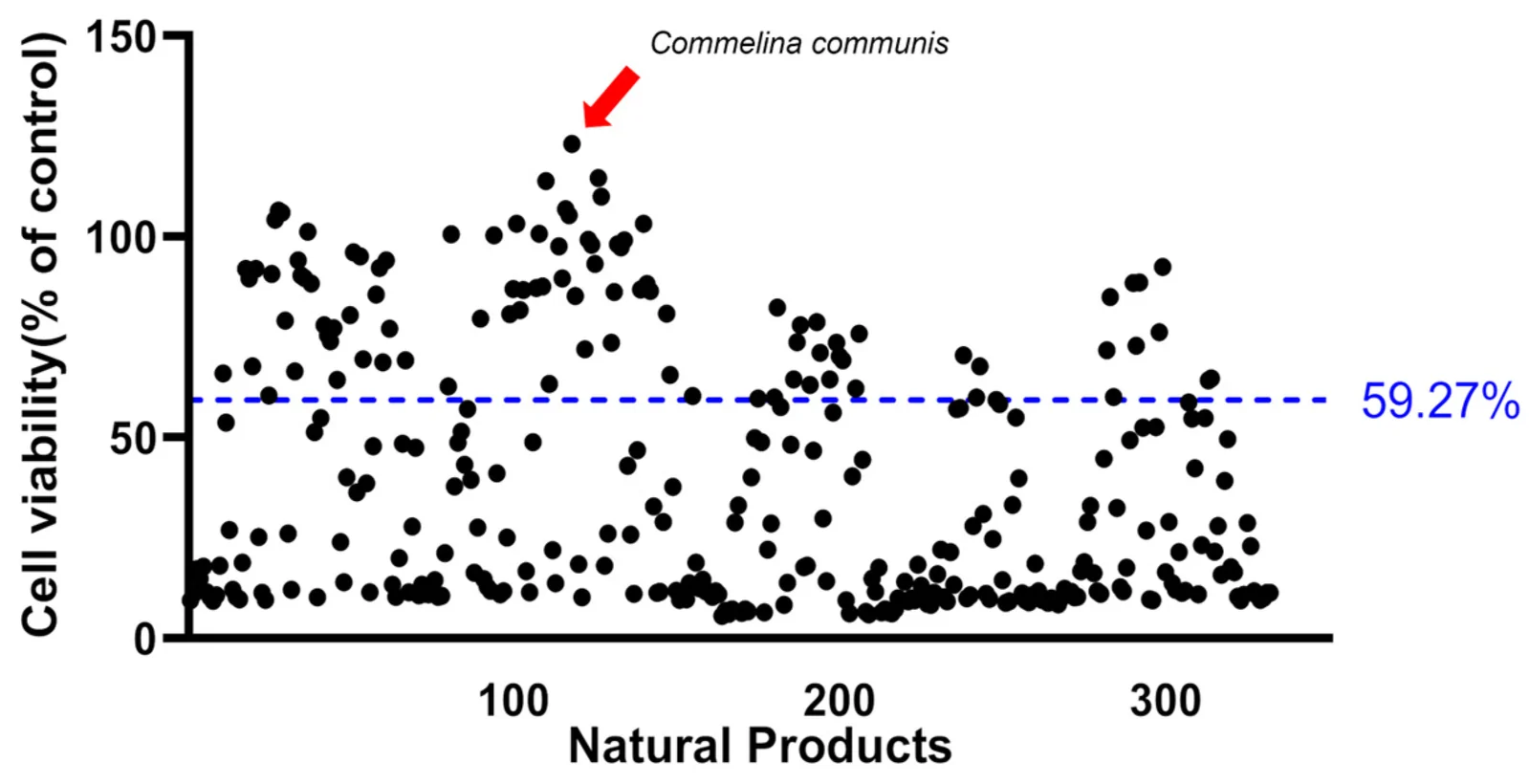
Natural products were screened to identify candidates capable of alleviating FB1-induced cytotoxicity in IPEC-J2 cells. The blue horizontal line represents the mean cell viability of the FB1-treated control wells across all screening plates, and each black dot represents the viability of cells co-treated with FB1 and an individual natural product.

A primary high-throughput screening of 332 natural product extracts was performed by co-treating IPEC-J2 cells with FB1 (25 μg/mL) and each extract (20 μg/mL) for 48 h (Figure 4). The extracts were ranked according to their ability to restore cell viability relative to the FB1-treated group. The six highest-ranking extracts—*Commelina communis*, *Aeschynomene indica*, *Elatine triandra*, *Cyperus glomeratus*, *Polygonum lapathifolium*, and *Scirpus tabernaemontani*—were selected for further evaluation (Table S1). The secondary validation results for the five candidates other than *C. communis* are presented in Figure S1. *C. communis* exhibited the highest cell viability in the primary screening and was therefore selected for further investigation. A non-cytotoxic concentration of *C. communis* was subsequently determined using the WST-1 assay, followed by evaluation of apoptosis-associated responses using Annexin V/PI staining and qRT-PCR.

### 2.5. Commelina communis Attenuates FB1-Associated Apoptotic Responses in IPEC-J2 Cells

**Figure 5:**
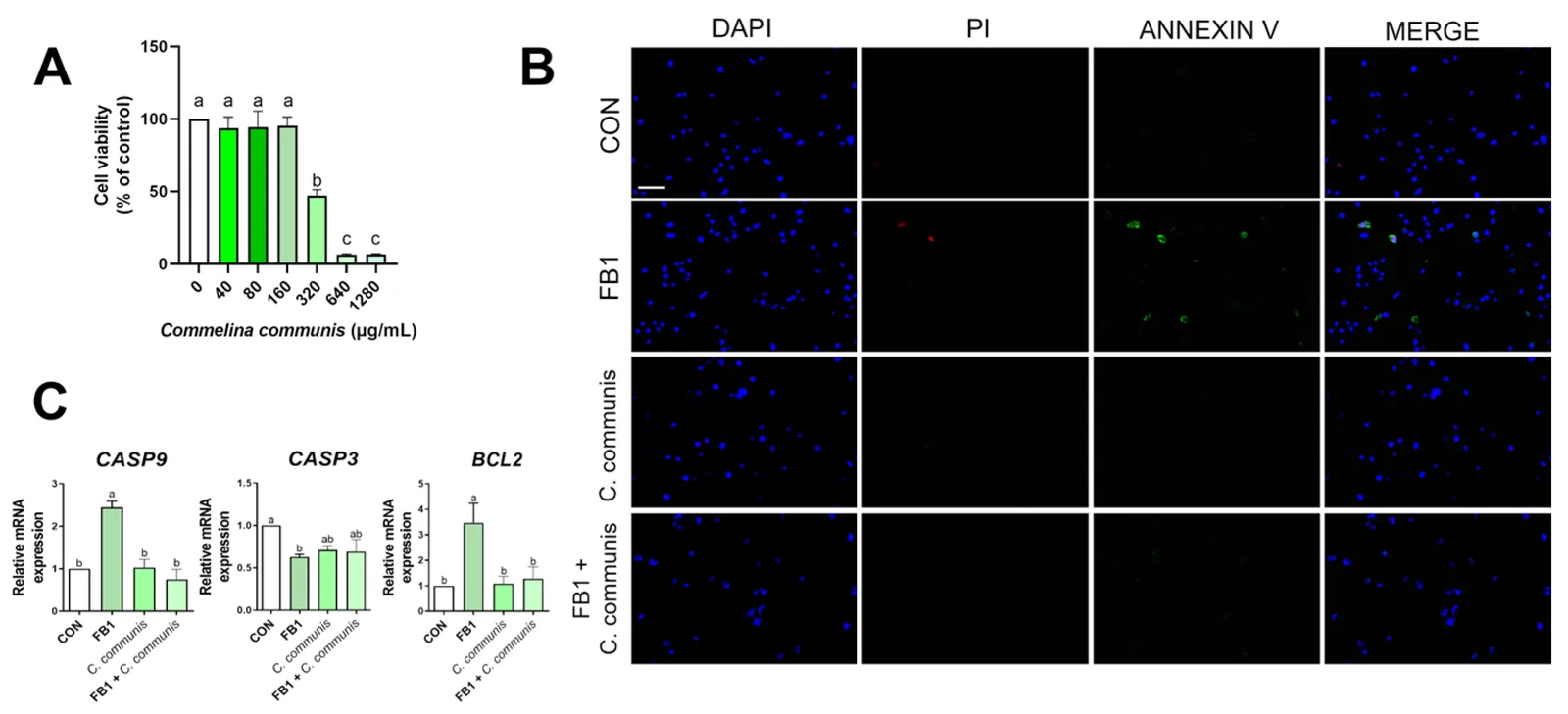
Effects of *C. communis* on FB1-associated apoptotic responses in IPEC-J2 cells. (**A**) Cell viability following *C. communis* treatment. (**B**) Representative Annexin V/PI fluorescence images following FB1, *C. communis*, or combined treatment. Scale bar = 150 μm. (**C**) mRNA expression of apoptosis-associated genes. Quantitative data in (**A**,**C**) are presented as the mean ± SEM from three independent experiments. Different letters indicate significant differences (*p* < 0.05).

Prior to evaluating its protective effects against FB1-induced toxicity, the cytotoxicity of *C. communis* was assessed in IPEC-J2 cells to determine the appropriate treatment concentration. Cell viability analysis showed that *C. communis* did not exert cytotoxic effects at the selected concentration, which was subsequently used for further experiments (Figure 5A). To examine apoptosis-associated changes, Annexin V/PI staining was performed. FB1 treatment was associated with an apparent increase in Annexin V/PI-positive staining, whereas co-treatment with *C. communis* reduced this staining pattern (Figure 5B). Additionally, the mRNA expression levels of apoptosis-related genes (*CASP9*, *CASP3*, and *BCL2*) were analyzed. qRT-PCR analysis showed that co-treatment with *C. communis* significantly counteracted FB1-induced alterations in *CASP9* (adjusted *p* = 0.0005) and *BCL2* (adjusted *p* = 0.0456) expression, whereas no significant difference in *CASP3* expression was observed between the FB1 and FB1 + *C. communis* groups (adjusted *p* = 0.9191) (Figure 5C). Together, these findings suggest that *C. communis* attenuates FB1-associated apoptotic responses in IPEC-J2 cells.

## 3. Discussion

FB1 was first isolated by Gelderblom et al. in 1988 [20]. It is known to contaminate various cereals, such as rice, barley, and wheat; however, it is mostly detected in corn. Because corn is a major component of swine feed, FB1 is considered a critical mycotoxin affecting pigs [4,5]. A previous study demonstrated that the oral administration of FB1 increases the expression of stress-related proteins in the small intestine and colon of pigs [21]. Another study found that FB1 impaired intestinal barrier function and induced cell cycle arrest in intestinal epithelial cells [5,13,22]. These findings suggest that FB1 exerts detrimental effects on the porcine gastrointestinal tract. However, a comprehensive understanding of the molecular interaction between FB1 and intestinal epithelial cells is lacking.

In this study, the cytotoxicity of FB1 in IPEC-J2 cells was initially evaluated using the WST-1 assay. Initial cell viability analysis showed an estimated IC_50_ value of 22.62 μg/mL for FB1. Accordingly, 25 μg/mL FB1 was used for the subsequent experiments. Exploratory gene expression profiling identified 1403 genes that met the predefined fold-change criterion, including 630 genes with increased expression and 773 genes with decreased expression. Subsequent GO and KEGG enrichment analyses of these candidate genes revealed enrichment of apoptosis-related biological processes. These findings suggested a potential association between FB1 exposure and apoptotic responses in intestinal epithelial cells.

Apoptosis is a highly regulated and evolutionarily conserved process that is essential for tissue homeostasis and animal development. The intrinsic (mitochondrial) apoptotic pathway is mediated by mitochondrial signaling, in which mitochondrial outer membrane permeabilization (MOMP) triggers the release of cytochrome c into the cytosol. This leads to the formation of the apoptosome and subsequent activation of *CASP9*, which, in turn, activates *CASP3* to execute apoptosis. Additionally, *BCL2*, a key anti-apoptotic regulator, plays an important role in maintaining mitochondrial membrane integrity and regulating intrinsic apoptotic signaling [22,23]. In this study, Annexin V/PI staining showed apoptosis-associated changes following FB1 exposure, while qRT-PCR analysis demonstrated significant alterations in the expression of *CASP9*, *CASP3*, and *BCL2*. These findings support the involvement of apoptotic responses in FB1-induced intestinal epithelial toxicity; however, additional protein-level and mitochondrial analyses are required to establish the specific apoptotic pathway involved.

A total of 332 natural products provided by the Nakdonggang National Institute of Biological Resources were evaluated to identify candidates capable of mitigating FB1-induced toxicity in porcine intestinal epithelial cells. Among these, *C. communis*, which exhibited the highest cell viability in the primary screening, was selected for further investigation. Annexin V/PI staining showed reduced apoptosis-associated staining in cells co-treated with *C. communis* and FB1 compared with cells treated with FB1 alone. Additionally, qRT-PCR analysis showed that co-treatment with *C. communis* partially counteracted FB1-induced alterations in the expression of apoptosis-related genes. These observations are consistent with attenuation of apoptosis-associated responses; however, they do not provide quantitative evidence of reduced apoptosis.

These findings provide a basis for further investigation of *C. communis* as a potential natural product for mitigating FB1-induced intestinal epithelial injury. However, several limitations should be acknowledged. Transcriptomic profiling was performed using one biological sample per condition and was therefore interpreted as exploratory rather than as a statistically powered differential expression analysis. In addition, Annexin V/PI staining was assessed qualitatively without quantification of apoptotic cell populations. Accordingly, the observed apoptosis-associated staining patterns and gene expression changes do not provide quantitative evidence of reduced apoptosis or confirmation of a specific apoptotic pathway. Although the origin, plant material information, extraction procedure, and storage conditions of the *C. communis* extract were documented, the extraction yield and chemical composition were not further characterized in the present study. In addition, the dose-dependent protective effect of *C. communis* against FB1-induced toxicity was not systematically evaluated. Further studies are therefore needed to characterize the chemical composition and bioactive constituents of the extract, establish dose–response relationships, clarify the underlying protective mechanisms, and validate its efficacy and safety in animal models.

## 4. Conclusions

In conclusion, FB1 exposure reduced IPEC-J2 cell viability, while exploratory transcriptomic profiling indicated FB1-associated expression changes involving apoptosis-related processes. High-throughput screening of 332 natural product extracts identified *Commelina communis* as a candidate for further evaluation. Co-treatment with *C. communis* was associated with reduced apoptosis-associated staining and partial counteraction of FB1-associated changes in apoptosis-related gene expression. These findings provide a basis for further investigation of *C. communis* and its bioactive constituents as potential modulators of FB1-induced intestinal epithelial injury. Further chemical characterization, mechanistic validation, and in vivo studies are required before its practical application can be established.

## 5. Materials and Methods

### 5.1. Cell Culture and Treatment

The IPEC-J2 cells used in this study were an established non-transformed porcine intestinal epithelial cell line (DSMZ ACC 701), originally isolated from the jejunal epithelium of a piglet. Cells were maintained in Dulbecco’s Modified Eagle Medium (DMEM) supplemented with 10% fetal bovine serum (FBS) and 1% penicillin–streptomycin at 37 °C in a humidified atmosphere containing 5% CO_2_. Fumonisin B1 (FB1; Sigma-Aldrich, St. Louis, MO, USA) was dissolved in dimethyl sulfoxide (DMSO) prior to treatment.

### 5.2. Cell Viability Analysis

IPEC-J2 cells were seeded in 24-well plates at a density of 5 × 10^3^ cells per well and treated with FB1. FB1 was applied at concentrations of 0, 5, 10, 25, and 50 μg/mL for 48 h. Following FB1 treatment, the viability of IPEC-J2 cells was evaluated using the water-soluble tetrazolium salt-1 (WST-1) assay. Absorbance values were subsequently recorded using a GloMax Discover Multi-Microplate Reader. The untreated control (0 μg/mL FB1) was used to normalize cell viability to 100% and was excluded from the nonlinear regression used to estimate the IC50. Nonlinear regression was performed using the non-zero FB1 concentrations (5, 10, 25, and 50 μg/mL). To evaluate the cytotoxicity of *C. communis*, IPEC-J2 cells were treated with *C. communis* at concentrations of 0, 40, 80, 160, 320, 640, and 1280 μg/mL for 48 h, and cell viability was measured using the WST-1 assay as described above. Based on these results, 160 μg/mL *C. communis* was selected for subsequent experiments.

### 5.3. Gene Expression Profiling

IPEC-J2 cells were treated with FB1 for 48 h prior to total RNA extraction. Total RNA was isolated using the AccuPrep Universal RNA Extraction Kit, and RNA quality was assessed using an Agilent 2100 Bioanalyzer with an RNA 6000 Nano Chip (Agilent Technologies, Amstelveen, The Netherlands). RNA concentration was measured using a NanoDrop ND-2000 Spectrophotometer (Thermo Fisher Scientific, Waltham, MA, USA). Libraries were constructed using the QuantSeq 3′ mRNA-Seq Library Prep Kit (Lexogen, Vienna, Austria) according to the manufacturer’s instructions. Briefly, 500 ng of total RNA was hybridized with an oligo-dT primer containing a partial Illumina adapter sequence, followed by reverse transcription and second-strand synthesis. The resulting cDNA libraries were purified, amplified to introduce complete adapter sequences, and subjected to single-end sequencing using an Illumina NextSeq 500 platform with a read length of 75 bp. Sequence reads were quality-trimmed using BBDuk with an average Q20 criterion and mapped to the *Sus scrofa* reference genome from the NCBI database using Bowtie2. Gene-level read counts were obtained using BEDTools. Read-count data were normalized using trimmed mean of M-values (TMM) and counts per million (CPM) normalization, and normalized expression values were provided on a log2 scale. Gene expression comparisons were performed using ExDEGA (Ebiogen, Seoul, Republic of Korea). Transcriptomic profiling was performed using one biological sample per condition and was considered exploratory. Genes showing a ≥1.5-fold increase or a ≤0.67-fold decrease in expression following FB1 treatment were selected for subsequent GO and KEGG enrichment analyses using DAVID.

### 5.4. Annexin V and Propidium Iodide Staining

Following 48 h of FB1 exposure, IPEC-J2 cells were harvested and washed with PBS. The cells were then centrifuged, the supernatant was removed, and the pellet was resuspended in 100 μL of 1× Annexin binding buffer.

The cell suspension was stained by adding 5 μL of Alexa Fluor 488 Annexin V (Thermo Fisher Scientific, Wilmington, DE, USA) and 1 μL of propidium iodide (PI) working solution (100 μg/mL). Samples were incubated for 15 min at room temperature in the dark. Thereafter, 3.5 μL of DAPI (Vector Laboratories, Burlingame, CA, USA) was added, and the stained cells were mounted on glass slides with coverslips. Fluorescence images were obtained using a fluorescence microscope (Korealabtech, Seongnam-si, Gyeonggi-do, Republic of Korea).

### 5.5. Quantitative Real-Time Polymerase Chain Reaction

IPEC-J2 cells were seeded into 35 mm culture dishes at a density of 2 × 10^5^ cells and allowed to grow for 48 h. Following this initial incubation, cells were exposed to 25 μg/mL of FB1 for an additional 48 h. Total RNA was extracted using the AccuPrep Universal RNA Extraction Kit (Cat. No. K-3141, Bioneer, Daejeon, Republic of Korea), and 1 µg of RNA was used to synthesize complementary DNA (cDNA) using the DiaStar™ RT Kit (SolGent, Daejeon, Republic of Korea). Primers for the genes of interest were designed using Primer3 (http://frodo.wi.mit.edu) and the primer sequences are provided in Table 1. Quantitative real-time PCR (qRT-PCR) was conducted under the following thermal cycling conditions: an initial denaturation at 95.0 °C for 3 min, followed by 40 amplification cycles of 95.0 °C for 15 s, 56.0–58.0 °C for 15 s, and 72.0 °C for 15 s. Gene expression levels were determined using the 2^−ΔΔCt^ method and normalized to glyceraldehyde-3-phosphate dehydrogenase (GAPDH) as the reference gene.

### 5.6. High-Throughput Screening Assay

A collection of 332 natural product extracts prepared with 70% ethanol was obtained from the Nakdonggang National Institute of Biological Resources (Sangju, Republic of Korea) and used for high-throughput screening. Each extract was supplied as a stock solution at 20 mg/mL. The *Commelina communis* L. extract used in this study was registered as resource no. FBCC-EP644. The plant material was collected on 16 August 2018, in Mungyeong-eup, Mungyeong-si, Gyeongsangbuk-do, Republic of Korea, and the extract was prepared from leaves using 70% ethanol. The six candidate extracts selected for secondary validation were prepared from dried plant materials using 70% ethanol at a sample-to-solvent ratio of 1 g:60 mL. The mixtures were subjected to ultrasound-assisted extraction using a bath-type sonicator (SD-120H, Sejin Ultrasonics, Seoul, Republic of Korea) at 40 kHz and 180 W for 30 min, followed by incubation at room temperature for 24 h. The extracts were then filtered, concentrated under reduced pressure, lyophilized, reconstituted in DMSO at 20 mg/mL, and stored at 4 °C until use. IPEC-J2 cells were seeded in 96-well plates at a density of 1 × 10^4^ cells per well and incubated for 24 h to allow attachment. For primary screening, cells were co-treated with FB1 (25 μg/mL) and each natural product extract (20 μg/mL) for 48 h. The primary screening was conducted once using five 96-well plates. Each natural product extract was tested in a single well, and each plate included one untreated control well and one FB1-treated control well for normalization and comparison of cell viability. The untreated control contained no DMSO, whereas the final DMSO concentration was maintained at 0.8% (*v*/*v*) among the FB1-containing screening groups by adjusting the vehicle volume with DMSO. Following treatment, 10 μL of EZ-Cytox (DoGenBio, Namyangju, Republic of Korea) was added to each well, and the plates were incubated at 37 °C for 2 h. Absorbance was measured using a GloMax Discover Multi-Microplate Reader (Promega, Madison, WI, USA), and cell viability was determined relative to the untreated control.

The extracts were ranked according to their ability to restore cell viability relative to the FB1-treated group, and the six highest-ranking extracts were selected for further evaluation. Before further evaluation, each selected extract was assessed for cytotoxicity to determine a non-cytotoxic treatment concentration. For the *C. communis* validation experiments, the untreated control contained no DMSO, whereas the final DMSO concentration was maintained at 1.5% (*v*/*v*) in the FB1-only, *C. communis*-only, and FB1 + *C. communis* groups. For *C. communis*, the WST-1 assay was used to select a non-cytotoxic concentration of 160 μg/mL, followed by Annexin V/PI staining and qRT-PCR. The secondary validation results for the five candidates other than *C. communis* are presented in Figure S1. Detailed information on the top 20 natural product extracts identified through the primary high-throughput screening is presented in Table S1.

### 5.7. Statistical Analyses

The primary high-throughput screening was performed once as an exploratory screen, whereas all subsequent validation experiments were independently repeated three times. Statistical analyses were performed using GraphPad Prism 8.4.2 for Windows (GraphPad Software, Boston, MA, USA). Data normality was assessed using the Shapiro–Wilk test, and homogeneity of variance was evaluated using the Brown–Forsythe test. Differences between two experimental groups were analyzed using Student’s *t*-test, whereas comparisons among more than two groups were performed using one-way ANOVA followed by Tukey’s multiple-comparison test. A *p*-value < 0.05 was considered statistically significant.

## Figures and Tables

**Table 1:** Gene-specific primer sequences used for qRT-PCR analysis.

Genes	Description	Accession No.	Sequence (5′-3′)	
*BEX5*	Brain expressed X-linked 5	XM_005657871.3	Forward	TAT CCT CAG CAG GTC CAC GT
			Reverse	CTT CTT CAT TCC GCA TTT GA
*TNNT1*	troponin T1	NM_213748.2	Forward	CGG AGG AGA AGA TGA GGA AG
			Reverse	TTT TAC GCT CGG ACA GAA TG
*AMY2*	amylase, alpha2B	NM_214195.1	Forward	GTG ATT GAT TTG GGT GGT GA
			Reverse	ATA GAT GCT CCC CCT GCT C
*CASP3*	caspase 3	NM_214131.1	Forward	CTC AGG GAG ACC TTC ACA AC
			Reverse	GCA CGC AAA TAA AAC TGC TC
*CASP9*	caspase 9	XM_013998997.2	Forward	TAC CCT GCC TTA CCT TCC AC
			Reverse	CTG GTC TTC GGT CAT CTG G
*BCL2*	BCL2 apoptosis regulator	XM_021099593.1	Forward	AAA GCC ACA AGG AGA AAA GC
			Reverse	TTC AGC CAC CGT AAA ATC TG
*GAPDH*	Glyceraldehyde-3-phosphate dehydrogenase	NM_001206359	Forward	ACA CCG AGC ATC TCC TGA CT
			Reverse	GAC GAG GCA GGT CTC CCT AA

## Data Availability

The original contributions presented in this study are included in the article and Supplementary Materials. Further inquiries can be directed to the corresponding author.

## Supplementary Materials

The following supporting information can be downloaded at: https://www.mdpi.com/article/10.3390/toxins18100445/s1, Table S1: Cell viability of the top 20 natural products identified through high-throughput screening; Figure S1: Validation of the five candidate natural products selected from high-throughput screening for alleviating FB1-induced toxicity in IPEC-J2 cells.

## Abbreviations

FB1	Fumonisin B1
IPEC-J2	Intestinal porcine epithelial cell line-J2
GO	Gene Ontology
KEGG	Kyoto Encyclopedia of Genes and Genomes
PI	Propidium iodide
WST-1	Water-soluble tetrazolium salt-1
qRT-PCR	Quantitative real-time polymerase chain reaction
