# Supplementary material for: Commelina communis Mitigates FB1-Induced Apoptotic Responses in Porcine Intestinal Epithelial Cells
Source: Toxins (Basel). 2026 Oct 15;18(10):445. doi: 10.3390/toxins18100445 (PMC13709597; doi:10.3390/toxins18100445)
Supplement: Supplementary file 1 [file toxins-18-00445-s001.zip › toxins-4587298-supplementary.pdf]

Supplementary Materials: *Commelina communis* mitigates FB1-induced apoptotic responses in porcine intestinal epithelial cells

Yo Han Lee, Ye Rin Bae, Tae Hong Kang, Jung Woong Yoon, Seung Joon Lim, Chae Hyun Lee, Suhyeon Yun, Sang In Lee

Table S1. Cell viability of the top 20 natural products identified through high-throughput screening.

NO.	Natural products	Solvent	Cell Viability(%)
1	Commelina communis	70% Ethanol	123.0426
2	Aeschynomene indica	70% Ethanol	114.524
3	Elatine triandra	70% Ethanol	113.8264
4	Cyperus glomeratus	70% Ethanol	109.9182
5	Polygonum lapathifolium	70% Ethanol	106.757
6	Scirpus tabernaemontani	70% Ethanol	106.4047
7	Ranunculus chinensis	70% Ethanol	105.9652
8	Juncus diastrophanthus	70% Ethanol	105.2878
9	Rorippa cantoniensis	70% Ethanol	104.1352
10	Monochoria korsakowii	70% Ethanol	103.2189
11	Nymphaea tetragona	70% Ethanol	103.1674
12	Carex miyabei	70% Ethanol	101.2419

13	Phragmites japonica	70% Ethanol	100.7495
14	Nasturtium officinale	70% Ethanol	100.5672
15	Anthriscus sylvestris	70% Ethanol	100.3056
16	Marsilea quadrifolia	70% Ethanol	99.21691
17	Impatiens textori	70% Ethanol	99.01507
18	Lobelia chinensis	70% Ethanol	98.11924
19	Lysimachia davurica	70% Ethanol	97.90555
20	Polygonum orientale	70% Ethanol	97.58469

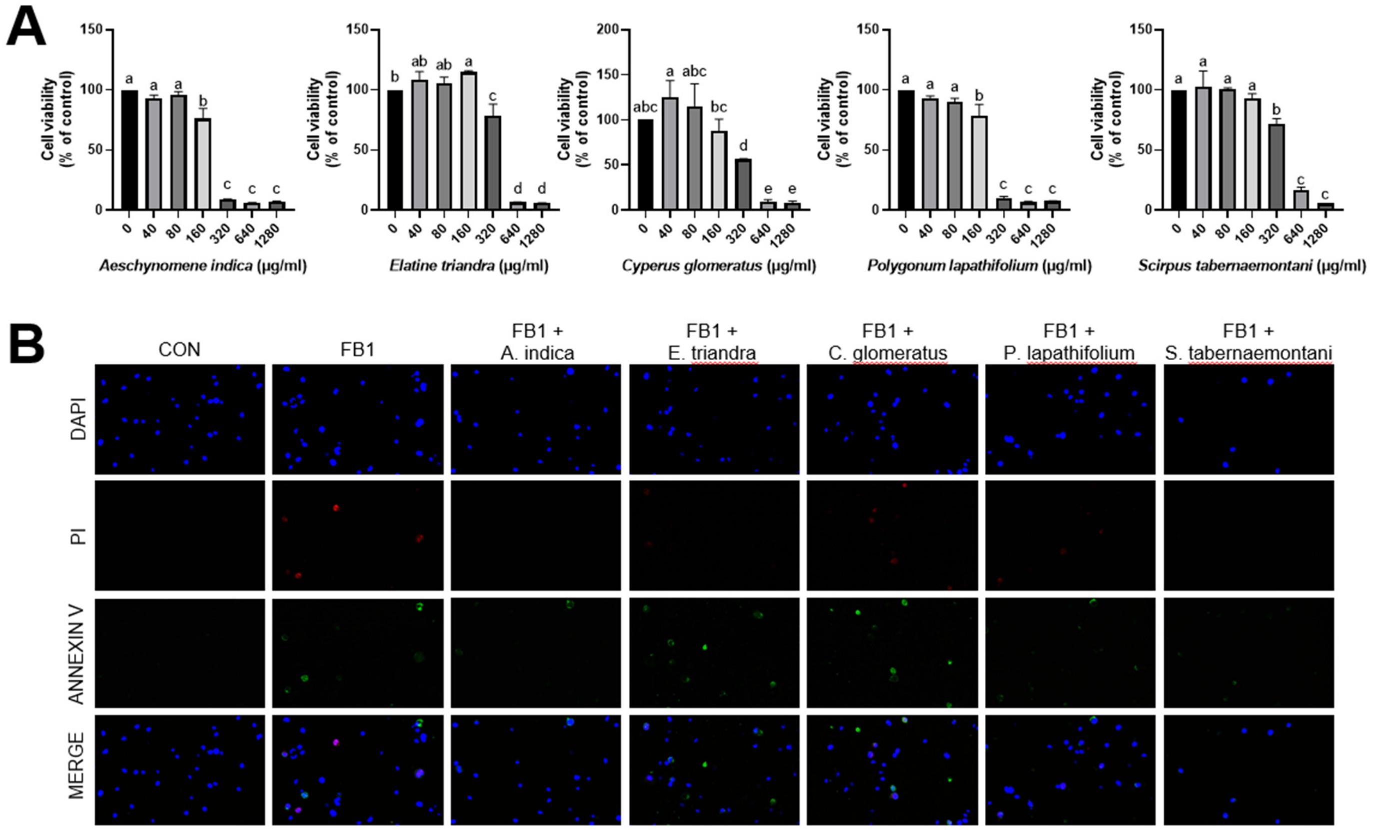

Figure S1: Validation of the five candidate natural products selected from high-throughput screening for alleviating FB1-induced toxicity in IPEC-J2 cells. (A) Cell viability of IPEC-J2 cells treated with different concentrations of *Aeschynomene indica*, *Elatine triandra*, *Cyperus glomeratus*, *Polygonum lapathifolium*, and *Scirpus tabernaemontani*. (B) Representative Annexin V/PI staining images of IPEC-J2 cells treated with FB1 alone or co-treated with FB1 and the indicated candidate natural products.
